# Taking stock: provider prescribing practices in the presence and absence of ACT stock

**DOI:** 10.1186/1475-2875-10-218

**Published:** 2011-08-03

**Authors:** Bernadette Hensen, Lucy Smith Paintain, Rima Shretta, Jane Bruce, Caroline Jones, Jayne Webster

**Affiliations:** 1 Clinical Research Department, London School of Hygiene and Tropical Medicine, WC1E 7HT, London, UK; 2 Department of Disease Control, London School of Hygiene and Tropical Medicine, WC1E 7HT, London, UK; 3 Center for Pharmaceutical Management, Management Sciences for Health, Arlington, VA, USA

## Abstract

**Background:**

Globally, the monitoring of prompt and effective treatment for malaria with artemisinin combination therapy (ACT) is conducted largely through household surveys. This measure; however, provides no information on case management processes at the health facility level. The aim of this review was to assess evidence from health facility surveys on malaria prescribing practices using ACT, in the presence and absence of ACT stock, at time and place where treatment was sought.

**Methods:**

A systematic search of published literature was conducted. Findings were collated and data extracted on proportion of patients prescribed ACT and alternative anti-malarials in the presence and absence of ACT stock.

**Results:**

Of the 14 studies identified in which ACT prescription for uncomplicated malaria in the public sector was evaluated, just six, from three countries (Kenya, Uganda and Zambia), reported this in the context of ACT stock. Comparing facilities with ACT stock to facilities without stock (i) ACT prescribing was significantly higher in all six studies, increasing by a range of 21.3% in children < 5 yrs weighing ≥ 5 kg (p < 0.001; Kenya 2006) to 51.7% in children ≥ 10 kg (p < 0.001; Zambia 2006); (ii) SP prescribing decreased significantly in five studies, by a range of 14.4% (p < 0.001; Kenya 2006), to 46.3% (p < 0.001; Zambia 2006); (iii) Where quinine was a reported alternative, prescriptions decreased in five of the six studies by 0.1% (p = 1.0, Kenya 2010) to 10.2% (p < 0.001; Zambia 2006). At facilities with no ACT stock on the survey day, the proportion of febrile patients prescribed ACT was < 10% in five of the nine target groups included in the six studies, with the proportion prescribed ACT ranging from 0 to 28.4% (Uganda 2007).

**Conclusions:**

Prescriber practices vary based on ACT availability. Although ACT prescriptions increased and alternative anti-malarials prescriptions decreased in the presence of ACT stock, ACT was prescribed in the absence, and alternative anti-malarials were prescribed in the presence of, ACT. Presence of stock alone does not ensure that treatment guidelines are followed. More health facility surveys, together with qualitative research, are needed to understand the role of ACT stock-outs on provider prescribing behaviours and preferences.

## Background

Despite progress in malaria control due to the availability of effective tools for treatment and prevention, access to interventions remains inequitable in sub-Saharan Africa, where an estimated 90% of the malaria-related mortality is concentrated, in part due to overstretched and under-resourced health systems [1]. Prompt and effective treatment of malaria episodes is fundamental to reducing morbidity and mortality [2]. The importance of treatment to the control of malaria is highlighted by various control initiatives and targets: the World Health Organization recommends treatment within 24 hours of symptom onset, particularly in children under five years of age, and the Roll Back Malaria (RBM) partnership established the target of diagnosing and treating 80% of malaria patients with an effective anti-malarial within 24 hours of illness onset by 2010 [3].

Traditionally, malaria treatment has relied on the use of monotherapies, firstly chloroquine and subsequently amodiaquine (AQ) and sulphadoxine-pyrimethamine (SP), as first-line therapy [4]. The WHO, following the development of widespread resistance to these monotherapies, has recommended the use of artemisinin-based combination therapy (ACT) as first-line treatment of uncomplicated malaria in sub-Saharan Africa since 2001. By 2009, all African countries had adopted ACT as the recommended first-line treatment policy [5,6]. Notwithstanding these policy changes, ACT coverage for episodes of febrile illness in children remains well below the 80% universal coverage target across Africa [7]. A summary of national level household survey data from 2007-2008 presented in the 2009 World Malaria Report highlights that ACT reached an average of only 15% of children under five years of age with a fever in the previous two weeks [7]. The RBM partnership reports coverage ranging from 0-50% amongst children living in African countries who received any anti-malarial treatment for malaria symptoms, based on administrative data from 2005 to 2009 [8].

Although highly efficacious, ACT is considerably more expensive than the monotherapies it replaces; therefore, parasitological diagnosis by microscopy or rapid diagnostic test (RDT) is now recommended to reduce misdiagnosis, ensure targeted treatment and prevent the development of resistance due to unnecessary drug pressure [9,10]. Procurement and supply chain management (PSM) procedures required to ensure ACT is delivered in the right quantity, to the right patient, at the right time, are the same as for other drugs used to treat malaria and other conditions. However, added complexities include: the availability of ACT in three to four different weight-specific dosing packages; the relatively short (two years) lifespan of the drug; and the recommendation of parasitological diagnosis using RDTs prior to treatment [11,12]. Consequently, ensuring the availability of these different components at public health facilities requires greater precision in quantification and supply management than for previous first-line treatments.

There are several processes contributing to the achievement of a successful coverage outcome which begin with treatment being sought for the febrile child. The source from which treatment is sought (for example, public sector health facilities, community delivery points, the retail sector, or traditional healers) influences the likelihood of whether or not a child will be prescribed or given an ACT [13]. Where parasitological diagnosis is available, treatment with ACT should be based upon the outcome of the test. However, in the absence of availability of parasitological diagnosis febrile children suspected of malaria should be given ACT unless there is a clear alternative diagnosis to malaria. Every febrile child accessing a public health facility where a) malaria parasites are identified, or b) no clear alternative diagnosis is made, should receive an ACT.

Globally, the monitoring of prompt and effective ACT treatment is conducted largely through household surveys. This coverage measure; however, provides no information on the facilitators of high coverage or the reasons for low coverage; in particular, the processes that occur at health facility or provider level are not investigated. The main processes in ACT delivery within the health facility are diagnosis, prescription, dispensing and communication between the health worker and the carer. To fully understand the malaria treatment pathway, health facility surveys are required to determine whether these processes are occurring. It is also important to investigate the potential predictors of effective delivery processes, which can be broadly categorised into characteristics of the health workers, characteristics of the children and their carer, and characteristics of the health facility.

Furthermore, assessments of prescribing and dispensing practices in the absence of data relating to ACT stock at the time and place of presumptive or parasitological diagnosis may underestimate the intent of health workers to prescribe recommended first-line treatment. Poor ACT coverage and a failure of healthcare workers to prescribe them may not always be attributable to lack of adherence to guidelines or failures of training programmes, but may also reflect the quality of the PSM or weaknesses within health systems. Assessments of prescribing practices and of the processes involved in treatment outcomes in the absence of stock information may lead to misguided assumptions and the development and implementation of interventions targeted at health workers that fail to demonstrate significant impact. The aim of this review is to assess evidence from health facility surveys on the extent to which prescribing of ACT and alternative anti-malarials in Africa varies in the presence and absence of stock of ACT.

## Methods

### Search strategy

A systematic search of published literature was conducted using the electronic database PUBMED (Medline). The review was conducted using various combinations of the following search terms: malaria, fever, anti-malarial, ACT, artemisinin-based, private sector, retail sector, community, health facility, quality of care, case-management, stock-out, shortage, stock, supply chain, distribution, prescrib*, dispens* (see Additional file 1 for full details). The final full search was conducted in June 2010. Grey literature was found by conducting internet searches for organizations managing malaria-related programmes, including ACTwatch, AMFm and Stop Stock-Outs. The final grey literature search was conducted in July 2010. All studies were imported into EndNote X3 for screening against the inclusion criteria.

### Inclusion criteria

Studies were considered eligible for inclusion based on the following criteria:

1) Disease of focus was uncomplicated malaria

2) Country of focus was in Africa

3) Country's first-line anti-malarial at the time of the study was an ACT

4) The study included an assessment of anti-malarial prescribing at the time treatment was sought

5) Prescribing practices were assessed in the context of ACT stock (with health facility surveys used to collect stock data)

Where intervention studies targeting provider practices or product supply management included a control group, data was only extracted for the control arm. Studies involving public or private health facilities were eligible for inclusion. Public facilities included government-run hospitals, health centres, health posts, NGO- or mission-run hospitals and health centres and community-based providers. The formal private sector included providers with professional qualifications and education, including doctors, clinical officers and nurses working for profit. Prescribing practices eligible for inclusion were practices for febrile patients with parasitologically-confirmed malaria or where a presumptive malaria diagnosis was made. It was assumed that where an anti-malarial was prescribed the diagnosis, whether presumptive or confirmed, was correct. Where a febrile patient was diagnosed parasitologically-negative, prescribing practices for these patients were not included in the analysis.

### Analysis of studies

For eligible studies, data relating to anti-malarial prescribing practices; ACT stock, frequency and duration of stock-outs; and analysis of ACT stock-out coping strategies (alternative anti-malarial prescribing practices) were extracted and imported into a results framework developed in Excel.

Provider prescribing behaviours were included only for presumptive or biologically-confirmed malaria cases that is anti-malarial prescribing practices for parasitologically-diagnosed malaria-negative patients was not considered.

Findings were collated and analysed to determine: 1) proportion of patients prescribed ACT at all facilities and stratified by presence or absence of ACT stock; 2) proportion of patients prescribed a non-ACT anti-malarial at all facilities and stratified by the availability (presence or absence) of ACT stock. The p-value and 95% confidence intervals for the differences between prescribing practices where analysis was restricted to facilities with stock compared with practices where analysis was restricted to facilities without stock on survey day were obtained using a chi-squared test in *R *version 2.11.1 (confidence intervals not adjusted for clustering at facility level).

## Results

**Figure 1:**
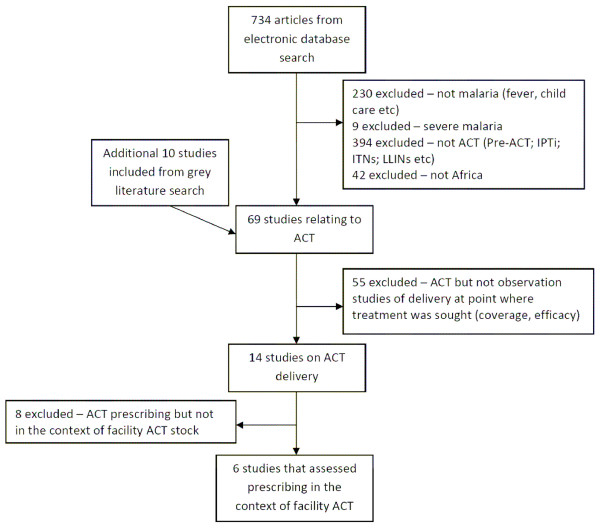
**Results of study inclusion process**.

The published literature search identified 734 studies and an additional ten reports were included from the grey literature. Of these, 14 studies assessed prescribing practices, but only six assessed prescribing practices in the context of facility ACT stock, and were thus included in the final analysis (Figure 1).

All six selected studies were cross-sectional studies conducted at public health facilities (see Additional file 2). Study procedures included exit interviews, clinical observations, facility surveys and health worker interviews [14-19]. Two studies were conducted in Kenya [14,19], one in Uganda [18] and three in Zambia [15-17]. All countries had adopted artemether-lumefantrine (AL) as their first-line ACT: Kenya in 2006, Uganda in 2004 and Zambia in 2002. Across the six studies prescribing practices were defined for nine populations: one study reported on prescribing for any age group [15], one presented data for children weighing ≥ 5 kg [19], two studies presented data based on age group (< 5 years or ≥ 5 years) [14,18] and two of the studies presented paediatric treatment data with respect to weight (< 5 kg and ≥ 5 kg) [16,17]. In this review, these nine populations were defined as the target groups in the analysis of results.

### ACT stock

Five of the six studies provided data on frequency and length of ACT stock-out (Additional file 2). One study provided no data on frequency or length of stock-out but presented prescribing practices at facilities regardless of ACT stock and at facilities with ACT stock [14]. The proportion of facilities with complete ACT stock out of all four weight-specific packs on the survey day ranged from 5.7% of facilities in Kenya in 2010 to 48.9% in Zambia in 2004 [16,17]. Three studies reported proportion of time spent with ACT stock-out, which ranged from approximately 30% of the year in a study from Zambia in 2006 to 38% of the time between October to December 2009 in Kenya [16,18].

### ACT prescribing at all facilities

Across facilities, regardless of ACT availability, the proportion of febrile patients that were prescribed an ACT ranged from 10.7% of children ≥ 10 kg in 2004 in Zambia to 66.4% of children < 5 yr in 2007 in Uganda [14,16] (Table 1). In 2006, four years after Zambia's malaria drug treatment policy change, a repeat cross sectional survey reported increased ACT prescribing practices at public health facilities for children ≥ 10 kg (10.7% to 42.2%; p < 0.001) compared with 2004 [16,17]. The highest proportion of the target group prescribed an ACT was reported at facilities in Kenya in 2010, six years after malaria treatment policy change, with 62.3% of children < 5 yrs and 63.5% of patients ≥ 5 yrs prescribed an ACT [14]. Similar relatively high levels of ACT prescribing were reported in Uganda in 2007, with 66.4% of children < 5 yrs and 56.2% of patients ≥ 5 yrs prescribed an ACT [18].

**Table 1:** ACT Prescribing at facilities with and facilities without ACT stock

Country, Year	% (No.) of patients diagnosed with uncomplicated malaria/fever and prescribed ACT			
	
	**At all facilities**, % (n)	**At facilities with ACT stock**, %(n)	**At facilities with no ACT stock**, %(n)	% difference^a^ (95% Confidence Interval^c^; p-value)
Kenya, 2006 [19]	26.4% (248/940)	28.1% (243/866)	6.8% (5/74)	**21.3%** (14.1%, 28.5%; p < 0.001)

Kenya, 2010 [14]	**< 5 yrs**	**< 5 yrs**	**< 5 yrs**	**< 5 yrs**
	
	62.3% (592/950)	64.1% (585/913)	18.9% (7/37)	**45.2%** (30.8%, 59.6%; p < 0.001)
	
	**≥ 5 yrs**	**≥ 5 yrs**	**≥ 5 yrs**	**≥ 5 yrs**
	
	63.5% (746/1175)	65.7% (730/1112)	25.4% (16/63)	**40.3%** (28.3%, 52.2%; p < 0.001)

Uganda, 2007 [18]	**< 5 yrs**	**< 5 yrs**	**< 5 yrs**	**< 5 yrs**
	
	66.4% (306/461)	69.4% (297/428)	27.3% (9/33)	**42.1%** (24.7%, 59.7%; p < 0.001)
	
	**≥ 5 yrs**	**≥ 5 yrs**	**≥ 5 yrs**	**≥ 5 yrs**
	
	56.2% (415/739)	60.3% (388/644)	28.4% (27/95)	**31.9%** (21.4%, 42.3%; p < 0.001)

Zambia, 2004 [16]	**≥ 10 kg**	**≥ 10 kg**	**≥ 10 kg**	**≥ 10 kg**
	
	10.7% (42/394)	21.9% (42/192)	0/202	**21.9%**

Zambia, 2006 [17]	**5-9 kg**	**5-9 kg**	**5-9 kg**	**5-9 kg**
	
	27.0% (149/552)	41.0% (144/351)	2.5% (5/201)	**38.5%** (32.6%, 44.5%; p < 0.001)
	
	**≥ 10 kg**	**≥ 10 kg**	**≥ 10 kg**	**≥ 10 kg**
	
	42.2% (231/547)	58.6% (219/374)	6.9% (12/173)	**51.7%** (44.9%, 58.3%; p < 0.001)

Zambia, 2006 [15]^b^	33.9% (494/1457)	44.7% (465/1040)	7% (29/417)	**37.7%** (33.7%, 41.8%; p < 0.001)

^a ^Difference is the proportion of patients who received ACT at facilities with stock minus the proportion of patients who received ACT at facilities with no ACT stock

^b ^Analysis restricted to facilities where diagnostics were available, test negative results excluded from analysis

^c ^Confidence interval not adjusted for clustering at facility level

### ACT prescribing in the presence and absence of stock

Where analysis of prescribing practices was restricted to facilities with ACT stock, prescribing increased across all studies compared to prescribing in all facilities regardless of the presence or absence of ACT stock. Where analysis was restricted to patients seen at facilities without ACT stock, ACT was still prescribed in eight out of the nine target groups (Table 1). However, estimates of the proportion of febrile patients prescribed ACT were lower in facilities with stock-outs of ACT in comparison with facilities with stock, with the proportion of patients prescribed ACT at facilities without stock ranging from 0% of children weighing ≥ 10 kg in Zambia 2004 to 28.4% of patients ≥ 5 yrs in Uganda 2007 [17,19].

Where analysis of prescribing practices was restricted to facilities with ACT and compared to ACT prescribing at facilities without ACT stock, a statistically significant increase in the proportion of febrile patients prescribed ACT was observed in all target groups across the six studies. The greatest difference in proportion of febrile patients prescribed ACT in facilities with ACT compared to facilities without ACT was 51.7% (58.6% to 6.9%; p < 0.001) for patients < 5 yrs, weighing ≥ 10 kg in the 2006 Zambian study [17]. A second study conducted in Zambia in 2006 showed a 37.7% increase in ACT prescribing where analysis was restricted to facilities with stock (44.7%) and compared to facilities without stock on the survey day (7%; p < 0.001) [15]. In studies conducted in Kenya in 2006 and 2010, Zambia in 2004 and in Uganda in 2007, there were similar significant differences in the proportion of febrile patients prescribed ACT when restricting analyses to facilities with ACT stock and comparing these estimates with analyses of prescribing at facilities without ACT stock [18,19].

### Alternative anti-malarial prescribing

Alternative anti-malarials were prescribed in all of the study sites, although these anti-malarials varied across countries and studies (Table 2, 3). Quinine (QN) was prescribed across all countries but prescriptions were low, at less than 10%. Other alternative anti-malarials prescribed and presented included artemether-lumefantrine+QN, AQ, SP, AQ+SP, chloroquine (CQ) and CQ+SP. Across all facilities the proportion of febrile patients prescribed any alternative anti-malarial ranged from 8.6% in Kenya in 2010 to 72.3% for children ≥ 10 kg in Zambia in 2004 (Table 2) [16,17]. Disaggregated by type of alternative anti-malarial, the proportion of patients prescribed an alternative ranged from 0.4% of children < 5 yrs prescribed SP in 2007 in Uganda [18] to 67.5% of children ≥ 10 kg prescribed SP in 2004 in Zambia [16] (Table 3).

**Table 2:** Alternative Anti-Malarial Prescribing in the Presence and Absence of ACT Stock

Country, Year	% (No.) of patients diagnosed with uncomplicated malaria/fever and prescribed Alternative (non-ACT) Anti-Malarial			
	
	**At all facilities**, % (n)	At facilities with ACT stock, % (n)	At facilities with no ACT stock, % (n)	% difference^a^ (95% Confidence Interval^c^; p-value)
Kenya, 2006 [19]	50.5% (475/940)	47.8% (414/866)	82.4% (61/74)	**-34.6%** (-44.6%, -24.6%; p < 0.001)

Kenya, 2010 [14]	8.6% (183/2125)	8% (162/2025)	21% (21/100)	**-13%** (-21.6%, -4.4%; p < 0.001)

Uganda, 2007 [18]	**< 5 yrs**	**< 5 yrs**	**< 5 yrs**	**< 5 yrs**
	
	19.5% (90/461)	16.4% (70/428)	60.6% (20/33)	**-44.2%** (-62.9%, -25.6%; p < 0.001)
	
	**≥ 5 yrs**	**≥ 5 yrs**	**≥ 5 yrs**	**≥ 5 yrs**
	
	26.7% (197/739)	22.5% (145/644)	54.7% (52/95)	**-32.2%** (-43.3%, -21.1%; p < 0.001)

Zambia, 2004 [16]	**≥ 10 kg**	**≥ 10 kg**	**≥ 10 kg**	**≥ 10 kg**
	
	72.3% (285/394)	56.8% (109/192)	87.1% (176/202)	**-30.3%** (-39.3%, -21.5%, p < 0.001)

Zambia, 2006 [17]	**5-9 kg**	**5-9 kg**	**5-9 kg**	**5-9 kg**
	
	43.5% (240/552)	24.2% (85/351)	77.1% (155/201)	**-52.9%** (-60.6%, -45.2%; p < 0.001)
	
	**≥ 10 kg**	**≥ 10 kg**	**≥ 10 kg**	**≥ 10 kg**
	
	34.6% (189/547)	17.4% (65/374)	71.7% (124/173)	**-54.3%** (-62.5%, -46.1%; p < 0.001)

Zambia, 2006 [15]^b^	36.6% (533/1457)	23.1% (240/1040)	70.3% (293/417)	**-47.2%** (-52.4%, -41.9%; p < 0.001)

^a ^Difference is the proportion of patients who received alternative anti-malarials at facilities with ACT stock minus the proportion of patients who received alternative anti-malarials at facilities with no ACT stock

^b ^Analysis restricted to facilities where diagnostics were available, test negative results excluded from analysis

^c ^Confidence interval not adjusted for clustering at facility level

**Table 3:** Alternative anti-malarial prescribing at facilities with and without ACT stock

Country, year of study	Percentage of patients diagnosed with uncomplicated malaria/fever and prescribed alternative anti-malarials to ACT			
	
	All facilities, % (n)	Facilities with ACT stock, %(n)	No ACT stock	% difference^a ^(95% Confidence Interval^b^; p-value)
Kenya, 2006 [19]	**AQ**	**AQ**	**AQ**	**AQ**
	
	38.6% (363/940)	36.7% (318/866)	60.8% (45/74)	**-24.1%** (-36.4%, -11.8%; p < 0.001)
	
	**SP**	**SP**	**SP**	**SP**
	
	4.4% (41/940)	3.2% (28/866)	17.6% (13/74)	**-14.4%** (-23.8%, 4.8%; p < 0.001)
	
	**AQ+SP**	**AQ+SP**	**AQ+SP**	**AQ+SP**
	
	2.8% (26/940)	3% (26/866)	0/74	**3%**
	
	**Other AM^c^**	**Other AM^c^**	**Other AM^c^**	**Other AM^c^**
	
	3.3% (31/940)	3.3% (29/866)	2.7% (2/74)	**0.6%** (0.3%, 11%; p = 1.0)
	
	**QN**	**QN**	**QN**	**QN**
	
	1.5% (14/940)	1.5% (13/866)	1.4% (1/74)	**0.1%** (0.16%, 46.6%; p = 1.0)

Kenya, 2010 [14]^e^	**AL+QN**	**AL+QN**	**AL+QN**	**AL+QN**
	
	<**5 yrs:**5.8% (55/950) ≥ **5 yrs:**2.6% (31/1175)	<**5 yrs:**6% (55/913) ≥ **5 yrs:**2.8% (31/1112)	<**5 yrs:**0/37 ≥ **5 yrs:**0/63	<**5 yrs: 6%** ≥ **5 yrs: 2.8%**
	
	**SP**	**SP**	**SP**	**SP**
	
	2.6% (56/2125)	1.9% (38/2025)	18% (18/100)	**-16.1%**(-24.2%, 8%; p < 0.001)
	
	**QN**	**QN**	**QN**	**QN**
	
	<**5 yrs:**2.6% (25/950) ≥ **5 yrs:**1.4% (16/1175)	<**5 yrs:**2.6% (24/913) ≥ **5 yrs:**1.3% (14/1112)	<**5 yrs:**2.7% (1/37) ≥ **5 yrs:**3.2% (2/63)	<**5 yrs:**-**0.1%** (-0.1%, 41.1%; p = 1.0) ≥ **5 yrs: -1.9%** (-3.6%, .09%; p = 0.2)

Uganda, 2007 [18]	**CQ+SP**	**CQ+SP**	**CQ+SP**	**CQ+SP**
	
	<**5 yrs:**7.6% (35/461) **≥ 5 yrs:**18% (133/739)	**< 5 yrs:**5.1% (22/428) **≥ 5 yrs:**14.8% (95/644)	**< 5 yrs:**39.4% (13/33) **≥ 5 yrs:**40.0% (38/95)	**< 5 yrs: -34.3%** (-81.4%, -45.8%; p < 0.001) **≥ 5 yrs: -25.2%** (-36.1%, -14.4%; p < 0.001)
	
	**CQ**	**CQ**	**CQ**	**CQ**
	
	**< 5 yrs:**2.2% (10/461) **≥ 5 yrs:**3% (22/739)	**< 5 yrs:**2.1% (9/428) **≥ 5 yrs:**2.5% (16/644)	**< 5 yrs:**3% (1/33) **≥ 5 yrs:**6.3% (6/95)	**< 5 yrs: -0.9%** (-31.1%, 0.1%; p = 0.5) **≥ 5 yrs: -3.8%** (-9.5%, 1.8%; p = 0.08)
	
	**SP**	**SP**	**SP**	**SP**
	
	**< 5 yrs:**0.4% (2/461) **≥ 5 yrs:**1.0% (7/739)	**< 5 yrs:**0.5% (2/428) **≥ 5 yrs:**0.9% (6/644)	**< 5 yrs:**0 (0/33) **≥ 5 yrs:**1.0% (1/95)	**< 5 yrs: 0.5%** **≥ 5 yrs: -0.1%** **(0.1%, 41.1%; p = 1)**
	
	**QN**	**QN**	**QN**	**QN**
	
	**< 5 yrs:**5.9% (27/461) **≥ 5 yrs:**2.8% (21/739)	**< 5 yrs:**5.1% (22/428) **≥ 5 yrs:**2.5% (16/644)	**< 5 yrs:**15.2% (5/33) **≥ 5 yrs:**5.3% (5/95)	< 5 yrs: -10.1% (-24.1%, 4%; p = 0.05) ≥ 5 yrs: **-2.8%** (-8%, 2.5%; p = 0.2)

	**Other AM^d^**	**Other AM^d^**	**Other AM^d^**	**Other AM^d^**
	
	**< 5 yrs:**3.5% (16/461) **≥ 5 yrs:**1.9% (14/739)	**< 5 yrs:**3.5% (15/428) **≥ 5 yrs:**1.9% (12/644)	**< 5 yrs:**3.0% (1/33) **≥ 5 yrs:**2.1% (2/95)	**< 5 yrs: 0.5%** (0.16%,48.9%; p = 1.0) **≥ 5 yrs: -0.2%** (0.19%, 8.2%; p = 0.7)

Zambia, 2004 [16]	**SP**	**SP**	**SP**	**SP**
	
	**≥ 10 kg**: 67.5% (266/394)	**≥ 10 kg:**53.7% (103/192)	**≥ 10 kg:**80.7% (163/202)	**≥ 10 kg: -27%** (-36.5%, -17.6%; p < 0.001)
	
	**QN**	**QN**	**QN**	**QN**
	
	**≥ 10 kg**: 4.8% (19/394)	**≥ 10 kg:**3.1% (6/192)	**≥ 10 kg:**6.4% (13/202)	**≥ 10 kg: -3.3%** (-8%, 1.4%; p = 0.2)

Zambia, 2006 [17]	**SP**	**SP**	**SP**	**SP**
	
	**5-9 kg:**38.8% (214/552) **≥ 10 kg:**27.6% (151/547)	**5-9 kg:**21.9% (77/351) **≥ 10 kg:**13.6% (51/374)	**5-9 kg:**68.2% (137/201) **≥ 10 kg:**57.8% (100/173)	**5-9 kg: -46.3%** (-54.4%, -38.1%; p < 0.001) ≥ **10 kg: -44.2%** (-52.7%, -35.6%; p < 0.001)
	
	**QN**	**QN**	**QN**	**QN**
	
	**5-9 kg:**4.7% (26/552) **≥ 10 kg:**7.0% (38/547)	**5-9 kg:**2.3% (8/351) **≥ 10 kg:**3.7% (14/374)	**5-9 kg:**9.0% (18/201) **≥ 10 kg:**13.9% (24/173)	**5-9 kg:**-**6.7%** (-11.3%, -2,%; p < 0.001) **≥ 10 kg:**-**10.2%** (-16.1%, -4.2%; p < 0.001)

Zambia, 2006 [15]^f, g^	**SP**	**SP**	**SP**	**SP**
	
	32.8% (479/1457)	20.4% (212/1040)	64% (267/417)	**-43.6%** (-41.3%, -31.4%; p < 0.001)
	
	**QN**	**QN**	**QN**	**QN**
	
	3.7% (54/1457)	3.7% (28/1040)	6.2% (26/417)	**-2.5%** (-5.3%, -0.7%; p = 0.003)

N/A Not applicable

^a ^Difference is the proportion of patients who received alternative anti-malarials at facilities with ACT stock minus the proportion of patients who received alternative anti-malarials at facilities with no ACT stock

^b ^Confidence interval not adjusted for clustering at facility level

^c^Other AM = Other Anti-malarial, includes DHA, AQ+QN, SP+QN, AL+QN, AQ+SP+QN

^d^Other AM = Other Anti-malarial, includes AQ+SP, AL+SP, AL+SP+CQ, QN+SP, AL+QN, AL+CQ

^e ^Results are prescribing practices for febrile patients,

^f ^Results based on all age groups,

^g ^Analysis restricted to facilities where diagnositcs were available, test negative results excluded from analysis

### Alternative anti-malarial prescribing in the presence and absence of ACT

Where analysis of prescribing practices was restricted to facilities with ACT stock, a decrease in alternative anti-malarial prescribing, compared to alternative anti-malarial prescribing at all facilities regardless of the presence or absence of ACT stock, was observed in all studies (Table 2). A decrease in the proportion of febrile patients prescribed QN was observed in six out of the nine target groups and for SP in seven out of eight target groups across the six studies (Table 3). Prescribing practices for AQ in Kenya 2006 CQ+SP and CQ for patients < 5 yrs in Uganda 2007 also decreased, albeit marginally for some target groups [14,18,19].

At facilities with no ACT stock, the proportion of febrile patients prescribed any alternative anti-malarials was generally significantly higher in comparison with facilities with ACT in stock on the day of the survey, increasing by a range of 13% for patients in Kenya in 2010 to 54.3% for patients ≥ 10 kg in Zambia in 2006 [16,18] (Table 2, 3). Where results were disaggregated by type of alternative anti-malarial, the exception to this was prescribing of QN in Zambia 2004 and Uganda 2007 for patients ≥ 5 years of age, of CQ, SP and other anti-malarials in Uganda in 2007, of QN, AQ+SP and other anti-malarials in Kenya in 2006 and of AL+QN and QN in Kenya in 2010 [14,16,18,19] (Table 3). The greatest difference in proportion of alternative anti-malarials prescriptions at facilities without ACT stock compared with facilities with stock was observed in the proportion of febrile patients prescribed SP; the greatest difference in proportion of febrile patients prescribed SP in facilities with and without ACT stock was 46.3% (21.9% to 68.2%; p < 0.001) for patients 5-9 kg and 44.2% (13.6% to 57.8%; p < 0.001) for patients ≥ 10 kg in Zambia in 2006 [17]. In Uganda in 2007, the proportion of febrile patients prescribed CQ+SP was higher in facilities that had no ACT stock than when compared to facilities with ACT stock, increasing by 34.3% (5.1% to 39.4%; p < 0.001) for children < 5 yrs and by 25.2% (14.8% to 40% p < 0.001) for patients ≥ 5 yrs [18]. Similarly, in Kenya in 2006, the proportion of febrile patients prescribed AQ was higher in facilities that had no ACT stock than at facilities with ACT stock, increasing by 24.1% (36.7% to 60.8%; p < 0.001) [19].

## Discussion

ACT stock-outs were a common occurrence across studies included in this review, ranging from 5.7% to 48.9% of facilities experiencing total ACT stock-out on the day of the survey; where percentage of time with ACT stock-out was reported, chronic problems were indicated with facilities in Zambia in 2006 reporting ACT stock out for 30% of the year and facilities in Kenya in 2010, 38% reporting total ACT stock out between October to December 2009 [16,18].

ACT prescribing for uncomplicated malaria was considerably below the RBM target of 80% across all studies. One could predict that if providers know there is no ACT stock in their facility they will be less likely to prescribe it and more likely to fall back upon the traditional mono-therapies from the pre-ACT era; however stock cannot wholly explain provider behaviour given that not all malaria cases in facilities with ACT in the studies were prescribed ACT and in some studies ACT prescription continued in facilities despite ACT stock-outs. Although absence of ACT stock provided an explanation for much of the non-ACT prescribing behaviour, alternative anti-malarials were prescribed where ACT was available, particularly in studies conducted in the early years of ACT policy adoption.

This review highlights that, regardless of stock, ACT prescription increased and prescription of alternative anti-malarials decreased the longer after ACT policy introduction the study was conducted. Zambia was among the first countries to adopt ACT as first-line treatment for malaria in 2002 [20]; repeat cross-sectional studies indicate that four years post-policy change, ACT prescribing practices had increased significantly. In Kenya, where ACT was adopted as first-line in 2006, the study conducted in 2010 reported much higher levels of ACT prescribing than the study conducted in the same year as the introduction of the new policy. These findings support evidence that initial adoption of new anti-malarial policy can be slow due to a multitude of factors [21,22], including provider perceptions and practices [23-25]; however, ultimately policy maturation can lead to improved operational implementation and increased policy adherence by providers [17,26].

Challenges associated with changing national drug policies and successful translation of policy to practice are not new [27,28]. Prior to the implementation of ACT as first-line treatment, many malaria patients failed to access anti-malarial treatment, with evidence that providers failed to prescribe them [26,29]. ACT is however, more expensive compared with traditional mono-therapies and available in four weight-specific packs, adding cost and complexity to PSM systems and hence making the successful transition to ACT as first-line therapy even more challenging [20]. These more complex PSM systems also place an increased burden on weaker health systems, where the infrastructure and capacity to manage drug procurement and supplies need to be strengthened to ensure effective and efficient ACT delivery. Failure to address weaknesses of health and PSM systems results in insufficient stock of essential medicines at point of care. Improving coverage of and access to essential medicines therefore relies not only on provider prescribing and dispensing practices but also on the health system and the quality of the PSM systems within it.

It is important to note here that the results of this review focus on prescription, which may not necessarily reflect the drug or dosage that is dispensed, and that stock levels are also likely to influence dispensary level outcomes. Within the studies included in this review, only one study evaluated healthcare providers' ACT dispensing practices in the presence and absence of weight-specific ACT packages [14]. Dispensing practices in this study in Kenya in 2010 suggest that providers that prescribe ACT are familiar with treatment guidelines: where ACT is out of stock, providers combine pills or cut packs to ensure the appropriate weight-specific dose is dispensed. Where ACT is available, these practices decrease [14].

Similarly, it should be noted that for the two studies that presented data according to the result of a malaria diagnostic test (Kenya, Zambia), the anti-malarial prescribing practices in the presence and absence of ACT were only considered for malaria-positive cases and for patients where no diagnostic test was performed but were prescribed an anti-malarial. This was done to maintain as much consistency with the other four studies where only patients with a malaria diagnosis were included, even though these were diagnosed presumptively. It was, therefore, assumed that all diagnosis was correct (whether presumptive or confirmed), and that no providers prescribed an anti-malarial for a non-malaria patient. It is acknowledged that this is an over-simplification of provider malaria diagnosis and treatment behaviour [30,31], however the primary objective of this paper is to understand the influence of ACT stock rather than diagnostic tests on prescribing behaviour.

There is a dearth of information on the factors associated with increased ACT prescribing; some evidence suggests that in-service ACT training, availability of wall charts and access to national guidelines are not significantly associated with decisions to prescribe ACT [17,18] while other studies report that in-service ACT training is significantly associated with ACT prescribing [19]. A qualitative study from Kenya documents fear of stock-outs as limiting ACT prescribing practices, with shortages of different dose packs and erratic supply causing health workers to ration the drug to those deemed to have greatest need or who seem most "deserving" [25]. In addition, availability of alternative anti-malarials while ACT supplies were inadequate confused health workers about when to prescribe ACT. Thus, even when ACT is in stock, both uncertainty around the continuity of ACT supply and the availability of alternative anti-malarials are factors that seem to influence provider practice. Multifaceted assessments of prescribing practices that quantitatively evaluate practices in operational settings and in the context of ACT stock, and which also collate qualitative evidence of the factors that influence providers' decision-making processes, would lead to a better understanding of practices and to the development of evidence-based interventions that improve treatment outcomes [32].

**Figure 2:**
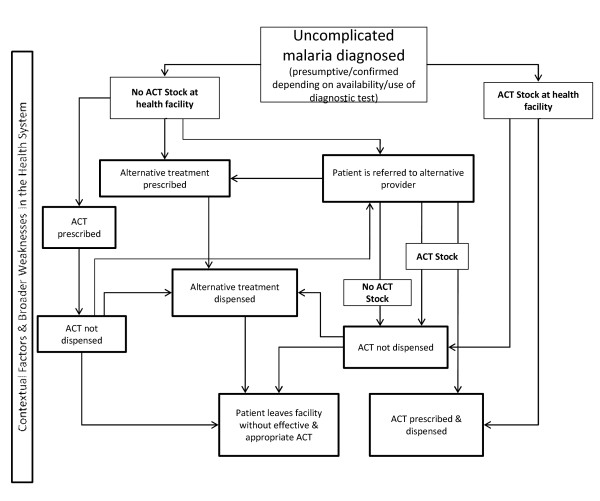
**Conceptual framework of prescribing and dispensing practices**.

To understand the processes involved in access to effective ACT treatment and provider coping strategies in the absence of ACT stock, we propose a framework of the treatment pathway in the presence and absence of ACT stock (Figure 2). The framework is supported by evidence derived from studies included in our review and highlights alternative prescribing and dispensing practices that providers may adopt. Understanding these practices, which healthcare providers adopt them and why would facilitate the development and implementation of appropriate interventions that contribute to improved ACT access and coverage.

This review is subject to limitations. Firstly, despite systematically searching published and unpublished literature, only a small number of studies from a single group of researchers were identified, conducted in only three African countries, providing a limited evidence base for discussion. The review is subject to language and publication bias and data may, therefore, exist that was not identified in the literature search. In addition, despite conducting internet searches for organizations managing malaria-related programmes, organizations were not contacted directly. There may, therefore, be grey literature that meets the inclusion criteria that was not identified in the search and therefore not included in the review. Cross-sectional surveys themselves have limitations when investigating prescribing practices since they only provide a "snapshot" in time and findings may differ if repeated after a relatively short period of time. As in the studies conducted in Kenya in 2006 and 2010, facility surveys were administered shortly after the nationwide distribution of AL [14,19]. Studies conducted at a different period in time may have presented very different outcomes. Finally, the analysis is subject to limitations: in the absence of the raw data sets variance estimation was not conducted, the analysis, therefore, does not adjust for clustering at the facility level. Despite these limitations, the findings relating to ACT prescribing practices are largely consistent in terms of direction; with ACT prescribing higher where stock is available and increased prescribing of alternative anti-malarials in the absence of ACT stock.

This review highlights the importance of quantifying the availability of ACT stock at the time of treatment seeking when assessing provider prescribing and dispensing practices as, in the absence of stock data, these practices are diluted. Facility surveys therefore, although not an appropriate methodology for stock monitoring, are valuable tools to assess prescribing and dispensing practices in the context of stock. Similarly, facility surveys which assess dose-specific stock may prove valuable in assessments of the accuracy of provider practices. Where reported, when ACT is prescribed and dispensed, the quality of prescribing and dispensing practices is high in studies included in our review with over 80% of patients prescribed ACT prescribed the recommended weight-specific ACT dose; in the context of weight-specific stock data, these practices may prove higher [14,17,19].

A greater understanding of the factors that influence providers' treatment decision-making process in the context of ACT stock and stock-outs is needed in order to direct interventions. Since 2009, the WHO has recommended the use of RDT prior to a malaria diagnosis, to reduce misdiagnosis, ensure targeted treatment and prevent the development of resistance [9,10]. RDTs not only add further complexity to the PSM of ACT, they add complexity to the diagnosis and treatment of malaria. Further research to understand provider prescribing and dispensing practices in the context of both the presence and absence of ACT and/or RDTs are, therefore, required. Finally, there is limited research regarding the effectiveness of referral to alternative providers and patient coping strategies in the absence of ACT stock. In a study from Kenya, over 30% of patients who sought care at public facilities failed to access drugs due to stock-outs at hospital pharmacies and more than 50% of patients issued ACT prescriptions to buy ACT outside of the hospital pharmacy did not purchase them [33]. Studies included in our review did not conduct a follow-up of patients to determine whether they were referred to alternative providers or whether patients purchased ACT at alternative locations using prescriptions provided and thus warrants further research. In addition, none of the studies we identified were conducted in the private sector. With a large proportion of malaria care-seeking directed at the private or community sectors, where access to ACT remain low, understanding the ACT prescribing practices of these providers also requires additional research [26].

## Conclusion

This review provides evidence of the influence of ACT stock and stock-outs on ACT and alternative anti-malarial prescribing practices. Where ACT is available, providers will prescribe them yet prescribing remains suboptimal. In addition, prescription of alternative anti-malarials continues, despite ACT availability. Provider prescribing practices in relation to treatment policy are not always rational and appropriate patient treatment cannot always be predicted. To adequately address inappropriate use of anti-malarials, multifaceted assessments of provider prescribing and dispensing practices are required. Patient-level treatment indicators assessed through household surveys, alone do not provide sufficient evidence to inform policy or programmes. The processes involved in malaria treatment and the context within which these processes take place must be evaluated in order for the outcomes to be fully understood and improved.

## Competing interests

The authors declare that they have no competing interests.

## Authors' contributions

BH carried out the literature search, collated the data and drafted the manuscript. LSP participated in the conception and design of the study and revised the manuscript critically. RS revised the manuscript for intellectual content. JB participated in the interpretation and analysis of data. CJ participated in the conception of the study and revised the manuscript critically for important intellectual content. JW conceived of the study, and participated in the design and coordination and revised the manuscript critically for intellectual content. All authors read and approved the final manuscript.

## Supplementary Material

Additional file 1 **Search strategy**. The key word and MeSH terms used to define the search strategy for the systematic review of published literature. Click here for file

Additional file 2 **Study characteristics**. Summary characteristics of the six studies included in the final analysis, including data on facility and study size, frequency and length of stock outs. Click here for file
