# Supplementary material for: Taking stock: provider prescribing practices in the presence and absence of ACT stock
Source: Malar J. 2011 Aug 3;10:218. doi: 10.1186/1475-2875-10-218 (PMC3163227; doi:10.1186/1475-2875-10-218)
Supplement: Additional file 1 — Search strategy. The key word and MeSH terms used to define the search strategy for the systematic review of published literature. [file 1475-2875-10-218-S1.DOC]

**Additional File** 1: Search Strategy

| **Search Engine** | Medline | **Search Terms** |
| --- | --- | --- |
|  |  | ACT* OR anti-malarial* OR artemisinin-based* |
|  |  |  |
|  |  | malaria* OR Malaria OR fever* OR Fever |
|  |  |  |
|  |  | Private sector* OR retail sector* OR community* |
|  |  |  |
|  |  | Case management^#^ OR Case management OR quality of care* OR quality of care |
|  |  |  |
|  |  | Prescrib* OR dispens* |
|  |  |  |
|  |  | Distribution* OR supply chain* |
|  |  |  |
|  |  | Health facility* OR Health facility |
|  |  |  |
|  |  | Stock-out OR shortage^#^ OR stock* |
|  |  |  |

* Keyword

^#^MeSH Terms
