# Supplementary material for: Taking stock: provider prescribing practices in the presence and absence of ACT stock
Source: Malar J. 2011 Aug 3;10:218. doi: 10.1186/1475-2875-10-218 (PMC3163227; doi:10.1186/1475-2875-10-218)
Supplement: Additional file 2 — Study characteristics. Summary characteristics of the six studies included in the final analysis, including data on facility and study size, frequency and length of stock outs. [file 1475-2875-10-218-S2.DOC]

**Additional File 2: Study c**haracteristics

| **Country, year of study** | **Survey period** | **Scale &**  **sector** | **Target group** | **# of patients included in study analysis** | **% (#) of consultations at facilities with ACT stock** | **% (#) of consultations at facilities without ACT stock** | **Cadres of healthcare provider interviewed**  **(%)** | **% (#) of facilities with total ACT stock-out on survey day** | **Median # days without any ACT in study facilities (% of time)** |
| --- | --- | --- | --- | --- | --- | --- | --- | --- | --- |
| Kenya, 2006[19] | Oct – Dec 2006 | 193 Public Facilities | Febrile patients <5yrs weighing ≥5kg | 940 | 92.1% (866/940) | 7.9%  (74/940) | N=227  Doctor (0.88%),  Clinical officer (19.8%), Nurse (66.1%),  Cadre without formal training (13.2%) | 12.4%  (24/193) | NR |
| Kenya, 2010[15] | Jan. – Feb. 2010 | 174 Public Facilities | Febrile, non-pregnant patients ≥5kg, <5yrs or ≥5yrs; with RDT or slide positive malaria or where no test performed | 2,125 | 95.3% (2025/2125)^a^ | 4.7%  (100/2125)^a^ | n=592  Doctor (2.4%),  Clinical Officer (23.1%), Nurses (69.8%), Clinically non-qualified staff (4.7%) | 5.7%  (10/174) | 35^b^  (38% of the period from Oct – Dec 2009) |
| Uganda, 2007[18] | May – Aug. 2007 | 188 Public, Private not-for-profit facilities | Febrile, non-pregnant patients >5kg, <5yrs or ≥5yrs | 1,200 | 89.3% (1072/1200) | 10.7%  (128/1200) | n=232  Doctor (1.3%),  Clinical officer (22.4%), Nurse (34.5%),  Midwife (3.9%),  Cadre without formal training (37.9%) | 13.3%  (26/195) | 60^c^  (33% of the 6 months prior the study period: May - August 2007) |
| Zambia, 2004[16] | Jan. – Mar 2004 | 94 Public Facilities | Febrile children <5yrs ≥10kg | 394 | 48.7% (192/394) | 51.3%  (202/394) | n=103  Clinical officer (29.1%)  Enrolled nurse (47.6%)  Registered nurse (3.9%) Nursing aid (19.4%) | 48.9%  (46/94) | NR |
| Zambia, 2006[17] | Mar – May 2006 | 104 Public Facilities | Febrile children <5yrs >5kg | 1,099 | 66% (725/1099) | 34%  (374/1099) | n=135  Clinical officer (28.2%)  Enrolled nurse (38.5%)  Registered nurse (7.4%)  Other (25.9%) | 40.4%  (42/104) | ~30% of the year (avg. no. of days with stock outs of different dose packages 108-123)^d^ |
| Zambia, 2006[14] | Mar – May 2006 | 76 Public Facilities | Sick outpatients with RDT or slide positive malaria or with no test performed with RDT or slide positive malaria or with no test performed | 1,457 | 71.4% (1040/1457) | 28.6%  (417/1457)^a^ | NR | NR^e^ | NR |

NR – Not Reported

^a^ %/No. of patients seen at facilities with ACT stock and where malaria diagnostic services were available

^b^ Median number of stock-outs days of all AL packs from Oct – Dec. 2009 (n=45)

Availability in the 6 months prior the survey; based on information from 157 facilities that had retrospective stock data available

^d^ Among 92 facilities with complete stock data

^e^ Study only reported number of consultations at facilities with/without ACT stock, not the number of facilities with/without ACT stock
